# Design and Synthesis of a Dual Linker for Solid Phase Synthesis of Oleanolic Acid Derivatives

**DOI:** 10.3390/molecules16064748

**Published:** 2011-06-08

**Authors:** Shaorong Wang, Weishuo Fang

**Affiliations:** State Key Laboratory of Bioactive Substances and Functions of Natural Medicines, Institute of Materia Medica, Chinese Academy of Medical Sciences and Peking Union Medical College, 1 Xian Nong Tan Street, Beijing 100050, China; Email: purplewinds@imm.ac.cn

**Keywords:** oleanolic acid, dual linker, solid phase synthesis, analytical construct

## Abstract

A hydrophilic amino-terminated poly(ethylene glycol)-type dual linker for solid phase synthesis of oleanolic acid derivatives using trityl chloride resin was designed and synthesized for the first time. Model reactions in both liquid and solid phase were performed to show the feasibility of its selective cleavage at two different sites. The biological assay results indicated that the long and flexible alkyl ether functionality in the linker is less likely to be critical for the binding event. Following the successful solid-phase synthesis of model compounds, the potential of this dual linker in reaction monitoring and target identification is deemed worthy of further study.

## 1. Introduction

**Figure 1:**
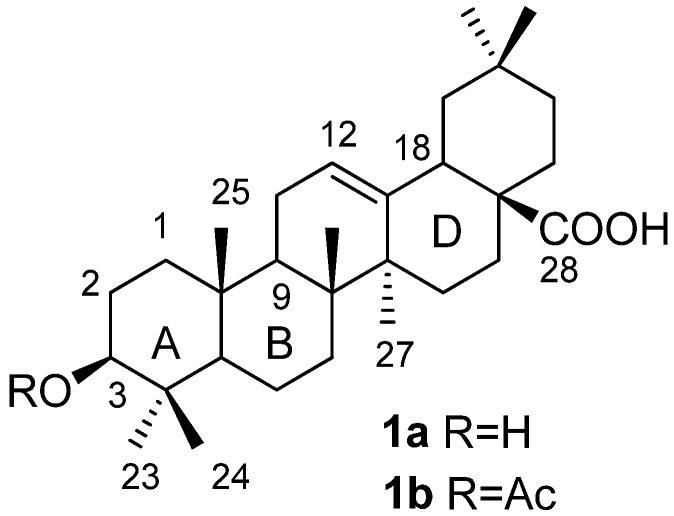
Structures of OA (**1a**) and 3β-acetoxyl-OA (**1b**).

While working on the modification of the A ring of oleanolic acid (OA, **1a**, Figure 1) to find novel antitumor compounds, we found that the C-28 carboxyl group must be protected during the acylation of C-3 OH, otherwise it would interfere the reaction. Organic synthesis on polymeric supports offers several advantages over solution-based techniques, including easy isolation of products by simple filtration from excess reagents after reaction completion, and it has been extensively used in the development of new hits based on natural products [1]. Although there is only few reports related to solid parallel synthesis of triterpenoids by attaching a resin to the C-28 position [2,3,4], we considered this methodology would not only serve as a means to protect the carboxyl group, but also could simplify the entire synthesis procedure. 

Since the OA scaffold was found to be highly unstable under harsh acidic conditions, trityl chloride resin, well known for its super sensitivity to acids, was chosen to facilitate the reaction. In addition, considering the steric hindrance around the C-28 position, a flexible anchor would be helpful for connecting the resin and the carboxyl group in order to improve the yield of the loading reaction. On the other hand, for the prospective target identification of an active OA analog, a reasonably designed linker pre-equipped from the start of the synthesis could help immobilizing the hit compounds onto solid matrix or an affinity tag rapidly without the need for laborious structure-activity relationship (SAR) studies [5], which would often be encountered in forward chemical genetics [6,7,8]. Based on these considerations, a novel dual linker between the triterpene scaffold and a trityl chloride polystyrene solid support was introduced, as far as we know, for the first time.

## 2. Results and Discussion

**Figure 2:**
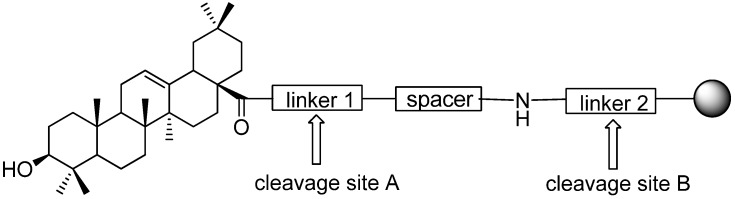
Diagram of the dual linker for OA solid phase synthesis.

Our designed dual linker also has an amino end, but our approach differs from that of a previous report [5], by directly attaching to the resin through an acid sensitive linker (linker 2, Figure 2). Cleavage at site B with a weak protonic acid will release the products bearing linker 1 and a hydrophilic spacer. This spacer could not only improve the resin’s swelling properties and the loading capacity as well as the overall yield of the solid phase reaction [9], but also exhibit more desirable physical properties, provide a friendly environment for target interaction and reduce the binding to nonspecific proteins in affinity chromatography for target identification [10]. In this initial study, we employed diethylene glycol to simply demonstrate the feasibility of the method. On the other hand, an alkoxymethyl ester fragment was chosen as linker 1 to protect the carboxyl group of the triterpene. It was supposed that the alkoxymethylene group would be sensitive to a Lewis acid like a 2-methoxy-ethoxymethyl ether (MEM), a commonly used alcohol protective group, so that facile cleavage at site A will yield the unlabeled product.

**Scheme 1:**
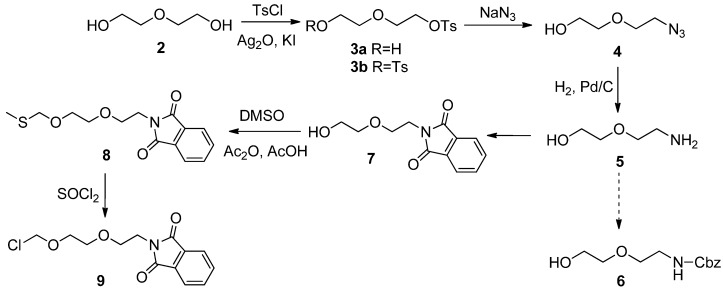
Synthesis of chloromethyl ether **9**.

The dual linker was synthesized as illustrated in Scheme 1. Monosulfonation of diethyleneglycol (**2**) with tosyl chloride (TsCl) in CH_2_Cl_2 _in the presence of pyridine and 4-dimethylaminopyridine (DMAP) showed poor selectivity towards the symmetrical diol, and yielded only a 48.9% of the monotosylate **3a**, along with 17.8% of the ditosylate **3b**, respectively. In contrast, use of a stoichiometric amount of TsCl in the presence of Ag_2_O and a catalytic amount of KI under neutral condition [11] improved the yield of the desired product **3a **to 90.6%. Then, **3a** was treated with NaN_3_ under reflux conditions in MeCN, giving the corresponding azide **4** in almost quantitative yield. A portion of DMF should be added to the system to accelerate the reaction process. Because of the high yield, compound **4** was hydrogenated in MeOH at atmospheric pressure (catalyzed by Pd/C) to give 2-(2-aminoethoxy)-ethan-1-ol (**5**) in 98.3% yield without further purification. 

We initially chose benzyloxycarbonyl chloride (Cbz) as the amino protective group before chloromethylation of the hydroxyl, but the yield is not satisfactory due to the poor selectivity of Cbz for the two nucleophiles, OH and NH_2_. Finally, we found that phthalimide group was the best choice to meet our needs, giving the protected compound **7** in a yield of 92.3%. Chloromethylation of **7** with trioxane and dry HCl (dissolved in dry MeOH) did not afford the wanted product but rather gave the dimer of the molecule itself by connecting with a methylene. Thus an *O*,*S*-acetal **8** was prepared in 83.7% yield by reacting of the alcohol **7** with DMSO in the presence of AcOH and Ac_2_O [12]. Then chloromethyl ether **9** was obtained by cleavage of **8** with a stoichiometric amount of SOCl_2_. It should be mentioned that **9** may be unstable and decompose on heating or during storage, so that it should be used immediately without any purification after evaporation of the reaction solvent at low temperature.

**Scheme 2:**
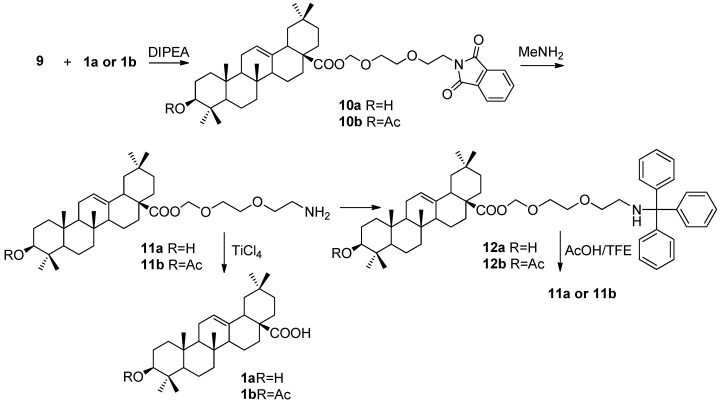
Dual cleavage property of the designed linker.

Before attempting the solid-phase experiment, preliminary liquid-phase tests were first performed on the assessment of dual cleavage ability of the linker (Scheme 2). Treatment of **1a** with **9** (~2 equiv) and *N*,*N*-diisopropylethylamine (DIPEA) (3 equiv) in CH_2_Cl_2_, furnished **10a** in 97.2% yield. The C-3 hydroxyl of **1a** is inert under this reaction condition. After hydrolysis of the phthalimide group in an ethanol solution of methylamine (33 wt. %), the amino product was obtained in 78.2% yield. Trityl chloride was chosen to simulate the resin, and we first attempted to couple it with **11a** through a carbamate, but were unsuccessful. Alternatively, a direct connection of the amine and trityl chloride in the presence of DIPEA proved effective, affording **12a** in a yield of 85.4%. Then, the cleavage conditions for the two different sites in **12a** were explored. It was found that a solution of 1% (v/v) trifluoroacetic acid (TFA) in CH_2_Cl_2 _could be used for removing the trityl group to afford **11a**, albeit accompanied with the formation of **1a**. Little improvement was achieved by reducing the concentration of TFA to 0.5%. Thus, a mixture of AcOH, 2,2,2-trifluoroethanol (TFE) and CH_2_Cl_2_ (1:2:7 v/v/v) was employed, giving solely the desired product **11a**. On the other hand, several Lewis acids, such as MgBr_2_, ZnBr_2_, AlCl_3_/*N*,*N*-dimethylaniline, *etc*, were utilized for the cleavage of the MEM-like moiety. Ultimately, anhydrous TiCl_4_ could smoothly release the free carboxyl group of OA without any side reactions after carefully controlling the reaction time. We also repeated the same reaction sequence starting from 3β-acetoxyl-OA **1b**. From the results, it was found that the finely tuned cleavage conditions of our designed dual linker did not affect the integrity of the triterpene skeleton and C-3 acetate.

**Scheme 3:**
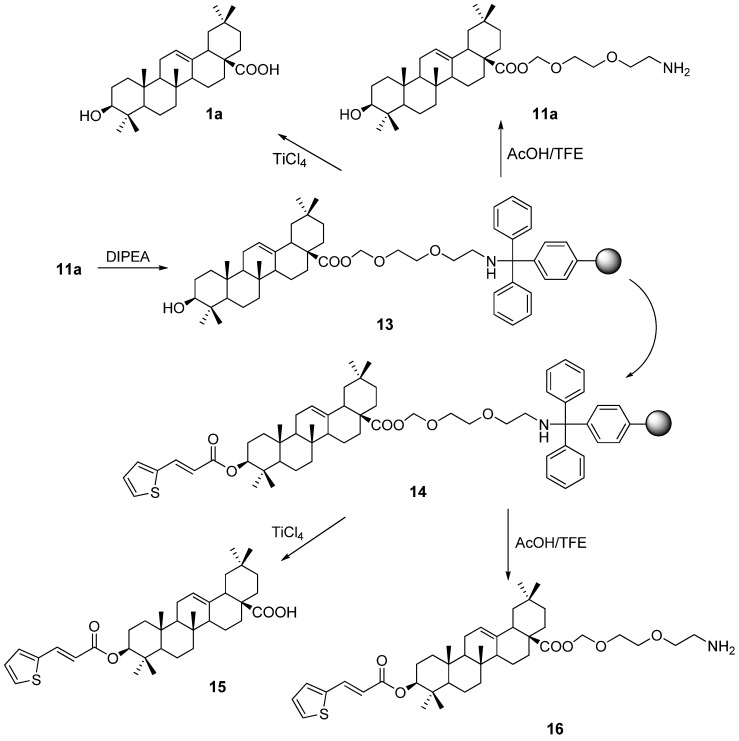
Dual cleavage property of the designed linker on the resin.

Compound **11a** was then loaded onto trityl chloride resin (Nankai HECHENG, 0.97 mmol/g) by procedures similar to those in solution described above. As indicated in Scheme 3, **11a** was incubated with pre-swelled resin (~2 equiv) in CH_2_Cl_2 _at room temperature in the presence of DIPEA. The reaction was quenched by MeOH as soon as the substrate disappeared in solution, as monitored by TLC. The loading of the substrate was calculated as 0.34 mmol/g (substrate/resin) based on the weight gain of the dried resin. Treatment of **13** with TiCl_4_ in CH_2_Cl_2 _easily recovered the free OA in 94.6%, while the treatment with AcOH/TFE could release the amino end from the resin in 93.5% smoothly. To show the viability of the dual linker for solid phase organic synthesis of derivatives of OA, a model reaction has been performed. The swelled resin **13** was mixed with excess *E*-(2-thiophene)acrylic acid, *N*,*N’*-diisopropylcarbodiimide (DIC) and DMAP in DMF. After 1 day, the reaction was terminated and the modified resin was filtered, dried and subjected to cleavage reactions under the conditions described above. The final products **15** and **16** were obtained in yields of 92.0% and 89.9%, respectively. The structures of the cleaved compounds **1a**, **11a**, **15**, and **16** were confirmed by MS, ^1^H- and ^13^C-NMR analysis.

Next, we measured and compared the cytotoxicity of two compounds, **1a** and **10a**, on non-small cell lung cancer cell line A549 (IC_50_ 51 μM and 50 μM, respectively), and their inhibition activity of human umbilical vein endothelial cells ECV304 (IC_50_ 20 μM and 10.3 μM, respectively). The results showed that both activity of these two compounds were on the same level, and indicated that the long and flexible alkyl ether functionality in the linker is less likely to be critical for the binding event, and the major binding interaction will come from the compound scaffold. This demonstrates its ability for application in the subsequent target identification program.

Since Merrifield and Tam first introduced the concept of “multidetachable” resins as early as 1979 [13,14], dozens of similar studies have been published [15,16,17,18,19]. Among these, a technique, named “analytical construct”, for solid phase reaction monitoring has been developed by Geysen *et al.* [20] and attracted much attention [21,22,23,24,25,26,27,28,29,30]. The construct typically comprises two selective-cleavable in-line linkers, involving a MS sensitizer (usually an amine) which can greatly improve the ionization properties of the materials and thus allowing easy detection by electrospray mass spectrometry [31] and a MS splitter which is an isotope label giving rise to a characteristic split-peak pattern in MS [20,32,33]. Accordingly, the amino-terminated dual linker designed by us may act as an analytical construct too, whose reaction monitoring ability is worthy of study in the near future.

## 3. Experimental

### 3.1. General

^1^H- and ^13^C-NMR spectra were measured on a Varian Mercury-400 or Varian Mercury-300 spectrometer. The mass spectra (MS) were measured on Agilent 1100 LC/MSD high performance ion trap mass spectrometer or LCQ ESI mass spectrometer.

### 3.2. Materials

Oleanolic acid was a reference compound (purity > 98%) purchased from the Division of Chinese Materia Medica and Natural Products, National Institute for the Control of Pharmaceutical and Biological Products (NICPBP), Ministry of Public Health, China. Trityl chloride resin (loading capacity 0.97 mmol/g) was purchased from Nankai HECHENG S&T Co. (Tianjing, China). All other reagents were of standard quality and used without further purification. All solvents were dried before use through standard procedures.

### 3.3. Tosylation of Diethyleneglycol *(**2**)*

To a stirred solution of diethyleneglycol (**2**, 0.1 mL, 1.0 mmol) in CH_2_Cl_2 _(10 mL) was added fresh Ag_2_O (350 mg, 1.5 mmol), TsCl (210 mg, 1.1 mmol), and KI (33 mg, 0.2 mmol). The reaction mixture was stirred at 0 °C for 4 h, then filtered through a small pad of silica gel, and washed with EtOAc. Evaporation of the solvent *in vacuo*, followed by column chromatography on silica gel (hexane-EtOAc = 1:1), gave the desired monotosylate product **3a** (as a colorless oil) and a spot of ditosylate **3b** (also a colorless oil) as a by-product.

*2-(2-{[(4-Methylbenzene)sulfonyl]oxy}ethoxy)ethan-1-ol* (**3a**) (242.5 mg, 90.6%): ^1^H-NMR (300 MHz, CDCl_3_): *δ* 7.78 (2H, d, *J* = 8.1 Hz, *o*-H of Ph), 7.33 (2H, d, *J* = 8.1 Hz, *m*-H of Ph), 4.17 (2H, t, *J* = 4.5 Hz, TsOCH_2_CH_2_O-), 3.66 (4H, m, -CH_2_CH_2_OCH_2_CH_2_-), 3.51 (2H, t, *J* = 4.5 Hz, -OCH_2_CH_2_OH), 2.43 (3H, s, -CH_3_), 2.18 (1H, s, -OH). ^13^C-NMR (75 MHz, CDCl_3_): *δ* 144.89, 132.87, 129.78 (2C), 127.86 (2C), 72.41, 69.12, 68.47, 61.52, 21.55. ESI-MS: *m/z* [M + H]^+^ 261.2, [M + Na]^+^ 283.1.

*2-(2-{[(4-Methylbenzene)sulfonyl]oxy}ethoxy)ethyl4-methylbenzene-1-sulfonate* (**3b**) (18.2 mg, 4.3%): ^1^H-NMR (300 MHz, CDCl_3_): *δ* 7.77 (4H, d, *J* = 8.1Hz, *o*-H of Ph), 7.34 (4H, d, *J* = 7.8 Hz, *m*-H of Ph), 4.08 (4H, t, *J* = 4.5 Hz, TsOCH_2_CH_2_O-), 3.59 (4H, t, *J* = 4.5 Hz, TsOCH_2_CH_2_O-), 2.44 (6H, s, -CH_3_). ^13^C-NMR (75 MHz, CDCl_3_): *δ* 144.91 (2C), 132.74 (2C), 129.83 (4C), 127.86 (4C), 68.96 (2C), 68.64 (2C), 21.56 (2C). ESI-MS: *m/z* [M + H]^+^ 415.1, [M + NH_4_]^+^ 432.1, [M + Na]^+^ 437.1, [M + K]^+^ 453.1.

### 3.4. Synthesis of 2-(2-azidoethoxy)ethan-1-ol *(**4**)*

To a stirred solution of **3a** (75.0 mg, 0.29 mmol) in dry CH_3_CN (2 mL) was added NaN_3_ (56.2 mg, 0.87 mmol) and DMF (200 μL). The reaction mixture was heated to reflux for 9 h, then another batch of NaN_3_ (56.2 mg, 0.87 mmol) was added to the system. After another 20 h, the reaction was quenched with water (2 mL), and extracted with CH_2_Cl_2_. The organic layer was dried over Na_2_SO_4_ and concentrated *in vacuo* to afford product **4** (36.4 mg, 96.2%) as a colorless oil. This compound was used in the next step without further purification. ^1^H-NMR (300 MHz, CDCl_3_): *δ* 3.76 (2H, t, *J* = 4.2 Hz,-OCH_2_CH_2_OH), 3.70 (2H, t, *J* = 4.8Hz, N_3_CH_2_CH_2_O-), 3.62 (2H, t, *J* = 4.2Hz, -OCH_2_CH_2_OH), 3.41 (2H, t, *J* = 4.8 Hz, N_3_CH_2_CH_2_O-), 2.05 (1H, br s, -OH). ^13^C-NMR (75 MHz, CDCl_3_): *δ* 72.38, 70.09, 61.78, 50.70. ESI-MS: *m/z* [M + NH_4_]^+^ 149.0.

### 3.5. Synthesis of 2-(2-aminoethoxy)ethan-1-ol *(**5**)*

A suspension of compound **4** (30 mg, 0.23 mmol), 10% Pd/C (10 mg) in MeOH (1 mL) was stirred under 1 atm of hydrogen pressure for 8 h, and then filtered. The filtrate was concentrated *in vacuo* to afford product **5** (23.6 mg, 98.3%) as a colorless oil. This compound was used in the next step without further purification. ^1^H-NMR (300 MHz, CDCl_3_): *δ* 3.64 (2H, t, *J* = 4.5Hz, -OCH_2_CH_2_OH), 3.49 (2H, t, *J* = 4.5 Hz, -OCH_2_CH_2_OH), 3.46 (2H, t, *J* = 5.1 Hz, H_2_NCH_2_CH_2_O-), 2.87 (3H, br s, -NH_2_ and -OH), 2.81 (2H, t, *J* = 5.1 Hz, H_2_NCH_2_CH_2_O-). ^13^C-NMR (75 MHz, CDCl_3_): *δ* 72.45 (2C), 61.16, 41.34. ESI-MS: *m/z* [M + H]^+^ 106.1, [M + Na]^+^ 128.1.

### 3.6. Synthesis of 2-[2-(2-hydroxyethoxy)ethyl]-2,3-dihydro-1H-isoindole-1,3-dione *(**7**)*

Compound **5** (1.05 g, 10 mmol) and phthalic anhydride (1.53 g, 10.1 mmol) were mixed together and stirred until the system become clear. Then the mixture was heated to 100 °C and maintained for 1.5 h. After cooling to room temperature, CHCl_3_ (15 mL) and water (6 mL) were added. The organic layer was dried over Na_2_SO_4_ and concentrated *in vacuo* to afford product **7** (2.17 g, 92.3%) as a white solid. This compound was used in the next step without further purification. ^1^H-NMR (300 MHz, CDCl_3_): *δ* 7.81 (2H, m, Ph), 7.69 (2H, m, Ph), 3.88 (2H, t, *J* = 5.4Hz, -NCH_2_CH_2_O-), 3.72 (2H, t, *J* = 5.4 Hz, -NCH_2_CH_2_O-), 3.66 (2H, t, *J* = 4.2Hz, -OCH_2_CH_2_OH), 3.57 (2H, t, *J* = 4.2Hz, -OCH_2_CH_2_OH), 2.51 (1H, br s, -OH). ^13^C-NMR (75 MHz, CDCl_3_): *δ* 168.37 (2C), 133.95 (2C), 131.93 (2C), 123.23 (2C), 72.13, 68.27, 61.63, 37.47. ESI-MS: *m/z* [M + H]^+^ 236.2, [M + Na]^+^ 258.1, [M + K]^+^ 274.1.

### 3.7. Synthesis of 2-(2-{2-[(methylsulfanyl)methoxy]ethoxy}ethyl)-2,3-dihydro-1H-isoindole-1,3-dione *(**8**)*

To a stirred solution of **7** (2.17 g, 9.2 mmol) in DMSO (27.1 mL) was added AcOH (5.4 mL,94.4 mmol) and Ac_2_O (17.9 mL, 191.4 mmol). The reaction mixture was stirred at room temperature for 20 h, then poured into a cold water (271 mL) solution of Na_2_CO_3_ (27.1 g). The mixture was extracted with CH_2_Cl_2_. The organic layer was dried over Na_2_SO_4_ and concentrated *in vacuo*. The residue was purified by column chromatography on silica gel (hexane-EtOAc = 5:1) to give the product **8** (2.28 g, 83.7%) as a viscous oil. ^1^H-NMR (300 MHz, CDCl_3_): *δ* 7.82 (2H, m, Ph), 7.69 (2H, m, Ph), 4.58 (2H, s, -SCH_2_O-), 3.89 (2H, t, *J* = 6.0 Hz, -NCH_2_CH_2_O-), 3.73 (2H, t, *J* = 6.0 Hz, -NCH_2_CH_2_O-), 3.63 (4H, br s, -OCH_2_CH_2_O-), 2.07 (3H, s, -SCH_3_). ^13^C-NMR (75 MHz, CDCl_3_): *δ* 168.17 (2C), 133.84 (2C), 132.07 (2C), 123.16 (2C), 75.32, 69.74, 67.80, 66.91, 37.16, 13.64. ESI-MS: *m/z* [M + H]^+^ 296.4, [M + Na]^+^ 318.4.

### 3.8. Synthesis of {2-[2-(1,3-dione-2,3-dihydro-1H-isoindol-2-yl)ethoxy]ethoxy}methyl olean-12-en-28-oate *(**10a**)*

To a stirred solution of **8** (51 mg, 0.17 mmol) in dry CH_2_Cl_2_ (1.0 mL) was added SOCl_2_ (12.3 μL, 0.17 mmol) at 0 °C. The reaction was warmed to room temperature and stirred for 2 h before it was evaporated under reduced pressure at 30 °C to remove the excess SOCl_2_. The crude product *2-{2-[2-(chloromethoxy)ethoxy]ethyl}-2,3-dihydro-1H-isoindole-1,3-dione* (**9**) could be used in the next step without further purification. The residue mentioned above dissolved in dry CH_2_Cl_2_ (0.5 mL) was added to a solution of the mixture of oleanolic acid (**1a**, 21.2 mg, 0.046 mmol) and DIPEA (21.3 μL, 0.13 mmol) in dry CH_2_Cl_2_ (0.5 mL). The mixture was stirred at room temperature for 2 h before it was diluted with CH_2_Cl_2_ (10 mL) and washed with a saturated solution of NaHCO_3_. The organic layer was dried over Na_2_SO_4_ and concentrated *in vacuo*. The residue was purified by column chromatography on silica gel (hexane-EtOAc = 3:1) to give the product **10a** (31.8 mg, 97.2%) as a white solid. ^1^H-NMR (400 MHz, CDCl_3_): *δ* 7.84 (2H, m, Ph), 7.72 (2H, m, Ph), 5.28 (1H, s, H-12), 5.24–5.18 (2H, ABq, *J* = 6.0 Hz, -COOCH_2_O-), 3.90 (2H, t, *J* = 6.0 Hz, -NCH_2_CH_2_O-), 3.74 (4H, m, -NCH_2_-, -OCH_2_CH_2_OCH_2_O-), 3.65 (2H, m,-OCH_2_CH_2_OCH_2_O-), 3.21 (1H, dd, *J* = 10.8, 4.4Hz, H-3α), 2.85 (1H, d, *J* = 10.8 Hz, H-18), 1.12 (3H, s, CH_3_), 0.98 (3H, s, CH_3_), 0.91 (3H, s, CH_3_), 0.89 (6H, s, CH_3_), 0.78 (3H, s, CH_3_), 0.72 (3H, s, CH_3_). ^13^C-NMR (100 MHz, CDCl_3_): *δ* 177.07, 168.20 (2C), 143.61, 133.91 (2C), 132.12 (2C), 123.22 (2C), 122.47, 89.37, 78.98, 69.62, 69.47, 68.00, 55.21, 47.59, 46.85, 45.83, 41.69, 41.17, 39.32, 38.73, 38.43, 37.15, 37.00, 33.81, 33.06, 32.74, 32.27, 30.66, 28.09, 27.58, 27.19, 25.81, 23.59, 23.39, 22.91, 18.31, 16.99, 15.56, 15.30. ESI-MS: *m/z* [M + Na]^+^ 726.4, [M + K]^+^ 742.4, [M + Cl]^−^ 738.6.

### 3.9. Synthesis of [2-(2-aminoethoxy)ethoxy]methyl olean-12-en-28oate *(**11a**)*

A mixture of **10a** (13.5 mg, 0.020 mmol) and a solution of methylamine in ethanol (0.7 mL, 33 wt. %) was stirred at room temperature for 0.5 h before it was concentrated *in vacuo*. The residue was purified by column chromatography on silica gel (CHCl_3_-MeOH = 10:1) to give the product **11a** (8.6 mg, 78.2%) as a white solid. ^1^H-NMR (400 MHz, CDCl_3_): *δ* 5.32–5.26 (3H, m, H-12, -COOCH_2_O-), 3.78 (2H, t, *J* = 4.4 Hz, -NCH_2_CH_2_O-), 3.62 (2H, t, *J* = 4.8 Hz, -OCH_2_CH_2_OCH_2_O-), 3.52 (2H, t, *J* = 5.2 Hz, -OCH_2_CH_2_OCH_2_O-), 3.21 (1H, dd, *J* = 12.4, 4.0 Hz, H-3α), 2.89-2.85 (3H, m, H-18,-NCH_2_CH_2_O-), 1.14 (3H, s, CH_3_), 0.98 (3H, s, CH_3_), 0.92 (3H, s, CH_3_), 0.90 (6H, s, CH_3_), 0.78 (3H, s, CH_3_), 0.74 (3H, s, CH_3_). ^13^C-NMR (75 MHz, CDCl_3_): *δ* 177.13, 143.49, 122.44, 89.21, 78.74, 72.21, 69.84, 69.29, 55.11, 47.48, 46.81, 45.71, 41.62, 41.22, 41.10, 39.24, 38.64, 38.37, 36.90, 33.71, 32.95, 32.66, 32.23, 30.57, 28.02, 27.50, 27.04, 25.73, 23.50, 23.30, 22.83, 18.22, 16.94, 15.53, 15.23. ESI-MS: *m/z* [M + H]^+^ 574.5, [M + Na]^+^ 596.7.

### 3.10. Synthesis of (2-{2-[(triphenylmethyl)amino]ethoxy}methyl olean-12-en-28-oate *(**12a**)*

To a solution of **11a** (63.4 mg, 0.11 mmol) in CH_2_Cl_2_ (2.0 mL) was added DIPEA (91.3 μL, 0.55 mmol) and trityl chloride (92.1 mg, 0.33 mmol). The reaction mixture was stirred at room temperature for 3 h before it was concentrated *in vacuo*. The residue was purified by column chromatography on silica gel (hexane-EtOAc = 6:1) to afford product **12a** (77.0 mg, 85.4%) as a white solid. ^1^H-NMR (400 MHz, CDCl_3_): *δ* 7.47 (6H, d, *J* = 7.6Hz, *o*-H of Ph), 7.27 (6H, t, *J* = 7.6 Hz, *m*-H of Ph), 7.18 (3H, t, *J* = 7.2 Hz, *p*-H of Ph), 5.30-5.22 (3H, m, H-12, -COOCH_2_O-), 3.73 (2H, t, *J* = 4.8 Hz, -NCH_2_CH_2_O-), 3.60 (2H, t, *J* = 5.2 Hz, -OCH_2_CH_2_OCH_2_O-), 3.53 (2H, t, *J* = 4.8 Hz,-OCH_2_CH_2_OCH_2_O-), 3.21 (1H, dd, *J* = 10.8, 4.4 Hz, H-3α), 2.86 (1H, dd, *J* = 13.6, 4.0 Hz, H-18), 2.36 (2H, br s, -NCH_2_CH_2_O-), 1.13 (3H, s, CH_3_), 0.98 (3H, s, CH_3_), 0.91 (3H, s, CH_3_), 0.90 (3H, s, CH_3_), 0.89 (3H, s, CH_3_), 0.77 (3H, s, CH_3_), 0.72 (3H, s, CH_3_). ^13^C-NMR (75 MHz, CDCl_3_): *δ* 177.04, 145.99 (3C), 143.53, 128.58 (6C), 127.69 (6C), 126.13 (3C), 122.41, 89.33, 78.83, 71.31, 70.51, 69.66, 69.31, 55.11, 47.50, 46.78, 45.74, 42.95, 41.62, 41.10, 39.24, 38.64, 38.34, 36.92, 33.71, 32.98, 32.68, 32.20, 30.57, 28.02, 27.52, 27.09, 25.75, 23.50, 23.30, 22.83, 18.22, 16.94, 15.50, 15.23. ESI-MS: *m/z* [M + H]^+^ 816.7.

### 3.11. Cleavage at Site A of ***12a*** with TiCl4

To a solution of **12a** (10.6 mg, 0.013 mmol) in CH_2_Cl_2_ (1.0 mL) was added TiCl_4_ (5 μL, 0.045 mmol) dropwise. The reaction mixture was stirred at room temperature for 0.5 h before it was poured into water (1 mL) and extracted with CH_2_Cl_2_. The organic layer was washed with a saturated solution of NaHCO_3_, dried over Na_2_SO_4_ and concentrated *in vacuo*. The residue was purified by column chromatography on silica gel (hexane-acetone = 5:1) to afford product **1a** (5.3 mg, 89.6%) as a white solid. ^1^H-NMR (300 MHz, pyridine-*d*_5_): *δ* 5.49 (1H, br s, H-12), 3.44 (1H, m, H-3α), 3.29 (1H, dd, *J* = 13.5, 3.6 Hz, H-18), 1.29 (3H, s, CH_3_), 1.24 (3H, s, CH_3_), 1.02 (9H, s, CH_3_), 0.95 (3H, s, CH_3_), 0.90 (3H, s, CH_3_). ^13^C-NMR (75 MHz, pyridine-*d*_5_): *δ* 180.20, 144.88, 122.61, 78.13, 55.88, 48.18, 46.72, 46.55, 42.23, 42.06, 39.82, 39.44, 39.01, 37.44, 34.29, 33.35 (2C), 33.24, 31.03, 28.85, 28.39, 28.16, 26.25, 23.89, 23.85, 23.76, 18.86, 17.49, 16.62, 15.63. ESI-MS: *m/z* [M−H]^−^ 455.3.

### 3.12. Cleavage at Site B of ***12a*** with TFE

A mixture of **12a** (25.0 mg, 0.031 mmol) and a solution of AcOH/TFE/CH_2_Cl_2_ (1.0 mL, v/v/v = 1:2:7) was stirred at room temperature for 2 h before it was quenched with a saturated solution of NaHCO_3 _(0.4 mL). The water layer was extracted with CH_2_Cl_2_, dried over Na_2_SO_4_ and concentrated *in vacuo*. The residue was purified by column chromatography on silica gel (CHCl_3_-MeOH = 10:1) to afford product **11a** (16.5 mg, 94.0%) as a white solid.

### 3.13. Synthesis of {2-[2-(1,3-dione-2,3-dihydro-1H-isoindol-2-yl)ethoxy]ethoxy}methyl 3β-acetoxylolean-12-en-28-oate *(**10b**)*

To a stirred solution of **8** (59 mg, 0.20 mmol) in dry CH_2_Cl_2_ (1.0 mL) was added SOCl_2_ (14.4 μL, 0.20 mmol) at 0 °C. The reaction was warmed to room temperature and stirred for 2 h before it was evaporated under reduced pressure at 30 °C to remove the excess SOCl_2_. The crude product **9** could be used in the next step without further purification. The crude product dissolved in dry CH_2_Cl_2_ (0.5 mL) was added to a solution of the mixture of 3β-acetoxyl-OA (**1b**, 22.9 mg, 0.046 mmol) and DIPEA (21.3 μL, 0.13 mmol) in dry CH_2_Cl_2_ (0.5 mL). The mixture was stirred at room temperature for 2 h before it was diluted with CH_2_Cl_2_ (10 mL) and washed with a saturated solution of NaHCO_3_. The organic layer was dried over Na_2_SO_4_ and concentrated *in vacuo*. The residue was purified by column chromatography on silica gel (hexane-EtOAc = 6:1) to give the product **10b** (32.7 mg, 95.4%) as a white solid. ^1^H-NMR (300 MHz, CDCl_3_): *δ* 7.82 (2H, m, Ph), 7.69 (2H, m, Ph), 5.25 (1H, br s, H-12), 5.23–5.15 (2H, ABq, *J* = 6.0 Hz, -COOCH_2_O-), 4.46 (1H, t, *J* = 8.1 Hz, H-3α), 3.88 (2H, t, *J* = 5.7 Hz, -NCH_2_CH_2_O-), 3.70 (4H, m, -NCH_2_-,-OCH_2_CH_2_OCH_2_O-), 3.62 (2H, t, *J* = 4.5 Hz, -OCH_2_CH_2_OCH_2_O-), 2.82 (1H, m, H-18), 2.02 (3H, s, CH_3 _of acetyl), 1.09 (3H, s, CH_3_), 0.89 (6H, s, CH_3_), 0.87 (3H, s, CH_3_), 0.84 (3H, s, CH_3_), 0.83 (3H, s, CH_3_), 0.70 (3H, s, CH_3_). ^13^C-NMR (75MHz, CDCl_3_): *δ* 176.99, 170.90, 168.12 (2C), 143.56, 133.86 (2C), 132.06 (2C), 123.16 (2C), 122.33, 89.27, 80.80, 69.55, 69.40, 67.93, 55.22, 47.43, 46.76, 45.73, 41.60, 41.10, 39.27, 38.03, 37.59, 37.07, 36.83, 33.74, 33.00, 32.60, 32.19, 30.58, 27.96, 27.49, 25.70, 23.53, 23.44, 23.32, 22.83, 21.23, 18.13, 16.91, 16.62, 15.29. ESI-MS: *m/z* [M + NH_4_]^+^ 763.5, [M + Na]^+^ 768.5.

### 3.14. Synthesis of [2-(2-aminoethoxy)ethoxy]methyl 3β-acetoxylolean-12-en-28-oate *(**11b**)*

A mixture of **10b** (129 mg, 0.17 mmol) and a solution of methylamine in ethanol (6.0 mL, 33 wt. %) was stirred at room temperature for 0.5 h before it was concentrated *in vacuo*. The residue was purified by column chromatography on silica gel (CHCl_3_-MeOH = 10:1) to give the product **11b** (86.9 mg, 82.0%) as a white solid. ^1^H-NMR (300 MHz, CDCl_3_): *δ* 5.28 (3H, m, H-12, -COOCH_2_O-), 4.48 (1H, t, *J* = 7.8 Hz, H-3α), 4.00 (2H, br s, -NH_2_), 3.77 (2H, m, -NCH_2_CH_2_O-), 3.63 (4H, m, -OCH_2_CH_2_O-), 3.02 (2H, m, -NCH_2_CH_2_O-), 2.86 (1H, m, H-18), 2.04 (3H, s, CH_3 _of acetyl), 1.13 (3H, s, CH_3_), 0.92 (6H, s, CH_3_), 0.90 (3H, s, CH_3_), 0.86 (3H, s, CH_3_), 0.85 (3H, s, CH_3_), 0.73 (3H, s, CH_3_). ^13^C-NMR (75 MHz, CDCl_3_): *δ* 177.05, 170.87, 143.47, 122.29, 89.15, 80.71, 72.56, 69.80, 69.26, 55.11, 47.34, 46.75, 45.62, 41.54, 41.31, 41.02, 39.18, 37.94, 37.51, 36.75, 33.67, 32.93, 32.54, 32.17, 30.54, 27.90, 27.42, 25.65, 23.47, 23.35, 23.26, 22.77, 21.20, 18.05, 16.88, 16.56, 15.26. ESI-MS: *m/z* [M + H]^+^ 616.4.

### 3.15. Synthesis of (2-{2-[(triphenylmethyl)amino]ethoxy}methyl 3β-acetoxylolean-12-en-28-oate *(**12b**)*

To a solution of **11b** (82.8 mg, 0.14 mmol) in CH_2_Cl_2_ (2.0 mL) was added DIPEA (155.5 μL, 0.95 mmol) and trityl chloride (188.3 mg, 0.68 mmol). The reaction mixture was stirred at room temperature for 3 h before it was concentrated *in vacuo*. The residue was purified by column chromatography on silica gel (hexane-EtOAc = 12:1) to afford product **12b** (82.3 mg, 71.3%) as a white solid.^ 1^H-NMR (300 MHz, CDCl_3_): *δ* 7.49 (6H, d, *J* = 7.5 Hz, *o*-H of Ph), 7.27 (6H, t, *J* = 7.2 Hz, *m*-H of Ph), 7.18 (3H, t, *J* = 7.2 Hz, *p*-H of Ph), 5.26 (3H, m, H-12, -COOCH_2_O-), 4.51 (1H, t, *J* = 7.8 Hz, H-3α), 3.74 (2H, t, *J* = 4.5 Hz, -NCH_2_CH_2_O-), 3.61 (2H, t, *J* = 4.8 Hz, -OCH_2_CH_2_OCH_2_O-), 3.53 (2H, t, *J* = 4.8 Hz, -OCH_2_CH_2_OCH_2_O-), 2.88 (1H, dd, *J* = 13.5, 3.3 Hz, H-18), 2.37 (2H, t, *J* = 4.8 Hz, -NCH_2_CH_2_O-), 2.05 (3H, s, CH_3 _of acetyl), 1.15 (3H, s, CH_3_), 0.93 (3H, s, CH_3_), 0.92 (3H, s, CH_3_), 0.89 (3H, s, CH_3_), 0.88 (3H, s, CH_3_), 0.87 (3H, s, CH_3_), 0.75 (3H, s, CH_3_). ^13^C-NMR (75 MHz, CDCl_3_): *δ* 177.05, 170.90, 146.04 (3C), 143.58, 128.61 (6C), 127.72 (6C), 126.18 (3C), 122.38, 89.36, 80.82, 71.35, 70.57, 69.70, 69.34, 55.23, 47.47, 46.82, 45.74, 42.99, 41.65, 41.15, 39.30, 38.06, 37.62, 36.86, 33.76, 33.01, 32.64, 32.25, 30.61, 27.99, 27.53, 25.73, 23.55, 23.46, 23.35, 22.86, 21.23, 18.16, 16.97, 16.63, 15.32. ESI-MS: *m/z* [M + H]^+^ 858.5, [M + Na]^+^ 880.5.

### 3.16. Cleavage at Site A of ***12b*** with TiCl4

To a solution of **12b** (25.0 mg, 0.030 mmol) in CH_2_Cl_2_ (1.0 mL) was added TiCl_4_ (22 μL, 0.20 mmol) dropwise. The reaction mixture was stirred at room temperature for 0.5 h before it was poured into water (1 mL) and extracted with CH_2_Cl_2_. The organic layer was washed with a saturated solution of NaHCO_3_, dried over Na_2_SO_4_ and concentrated *in vacuo*. The residue was purified by column chromatography on silica gel (hexane-EtOAc = 4:1) to afford product **1b** (12.0 mg, 82.8%) as a white solid. ^1^H-NMR (300 MHz, CDCl_3_): *δ* 5.27 (1H, br s, H-12), 4.49 (1H, t, *J* = 7.8 Hz, H-3α), 2.81 (1H, dd, *J* = 13.5, 4.2 Hz, H-18), 2.04 (3H, s, CH_3 _of acetyl), 1.12 (3H, s, CH_3_), 0.94 (3H, s, CH_3_), 0.92 (3H, s, CH_3_), 0.90 (3H, s, CH_3_), 0.86 (3H, s, CH_3_), 0.85 (3H, s, CH_3_), 0.74 (3H, s, CH_3_). ^13^C-NMR (75 MHz, CDCl_3_): *δ* 184.15, 171.01, 143.58, 122.53, 80.89, 55.27, 47.53, 46.52, 45.80, 41.51, 40.87, 39.25, 38.03, 37.67, 36.96, 33.76, 33.04, 32.49, 32.42, 30.65, 28.02, 27.64, 25.88, 23.56, 23.50, 23.36, 22.85, 21.29, 18.14, 17.15, 16.63, 15.37. ESI-MS: *m/z* [M−H]^−^ 497.3.

### 3.17. Cleavage at Site B of ***12b*** with TFE

A mixture of **12b** (26.6 mg, 0.031 mmol) with a solution of AcOH/TFE/CH_2_Cl_2_ (1.0 mL, v/v/v = 1:2:7) was stirred at room temperature for 2 h before it was quenched with a saturated solution of NaHCO_3 _(0.4 mL). The water layer was extracted with CH_2_Cl_2_, dried over Na_2_SO_4_ and concentrated in vacuo. The residue was purified by column chromatography on silica gel (CHCl_3_-MeOH = 10:1) to afford product **11b** (14.3 mg, 92.7%) as a white solid.

### 3.18. Loading of ***11a*** onto Trityl Chloride Resin

The trityl chloride resin (332.4 mg, 0.32 mmol) was swelled in CH_2_Cl_2_ (1 mL) for 1 h. Then a solution of **11a** (98.8 mg, 0.17 mmol) was added to the system, followed by DIPEA (177.8 μL, 1.21 mmol) dropwise. The reaction mixture was stirred at room temperature for 16 h. The system was treated with MeOH (1 mL) to cap unloaded resin. The resin was filtered and washed sequentially with CH_2_Cl_2_, DMF, CH_2_Cl_2_, diethyl ether and dried *in vacuo* for 24 h, affording resin **13 **(406.2 mg). The loading of the substrate was calculated as about 0.34 mmol/g (substrate/resin) based on the weight gain of the resin.

### 3.19. Acidic Cleavage at Site A of Resin ***13*** with TiCl4

Resin **13** (36.0 mg, 0.012 mmol) was swelled in CH_2_Cl_2_ (1 mL) for 1 h. TiCl_4_ (8.2 μL, 0.060 mmol) was added dropwise. The reaction mixture was stirred at room temperature for 0.5 h before the resin was filtered and washed with MeOH and CH_2_Cl_2_. The filtrate was concentrated *in vacuo* to afford product **1a** (5.3 mg, 94.6%) without any by-products.

### 3.20. Acidic Cleavage at Site B of Resin ***13*** with TFE

Resin **13** (39.5 mg, 0.013 mmol) was mixed with a solution of AcOH/TFE/CH_2_Cl_2_ (1.0 mL, v/v/v = 1:2:7) and the mixture was stirred at room temperature for 2 h before the resin was filtered and washed with MeOH and CH_2_Cl_2_. The filtrate was concentrated *in vacuo*, and the residue was purified by column chromatography on silica gel (CHCl_3_-MeOH = 10:1) to afford product **11a** (7.2 mg, 93.5%).

### 3.21. Coupling Reaction of (E)-(2-thiophene)acrylic Acid with Resin ***13***

Resin **13** (88.8 mg, 0.030 mmol) was swelled in DMF (1 mL) for 3 h. Then, (*E*)-(2-thiophene)acrylic acid (268.7 mg, 1.74 mmol), DMAP (21.3 mg, 0.17 mmol) and DIC (381.9 μL, 2.44 mmol) were added to the system. The reaction mixture was stirred at room temperature for 24 h before the resin was filtered, washed sequentially with DMF, MeOH, CH_2_Cl_2_, diethyl ether and dried *in vacuo* for 24 h, affording resin **14 **(92.6 mg). The conversion of the substrate was calculated as 93.3% based on the weight gain of the resin.

### 3.22. Acidic Cleavage at Site A of Resin ***14*** with TiCl_4_

Resin **14** (42.3 mg, 0.0137 mmol) was swelled in CH_2_Cl_2_ (1 mL) for 1 h. TiCl_4_ (9.6 μL, 0.085 mmol) was added dropwise. The reaction mixture was stirred at room temperature for 0.5 h before the resin was filtered and washed with MeOH and CH_2_Cl_2_. The filtrate was concentrated *in vacuo*, and the residue was purified by column chromatography on silica gel (hexane-acetone = 10:1) to afford product **15** (6.4 mg, 92.0%) and unreacted substrate **1a** (0.5 mg).

*3β-[(E)-(2-Thiophene)acryloxyl]-12-en-28-oate* (**15**): ^1^H-NMR (400 MHz, CDCl_3_): *δ* 7.76 (1H, d, *J* = 15.6 Hz, -CH = CHCO-), 7.36 (1H, d, *J* = 4.8 Hz, H-3 of thiophene), 7.25 (1H, d, *J* = 3.2 Hz, H-5 of thiophene), 7.05 (1H, t, *J* = 4.0 Hz, H-4 of thiophene), 6.23 (1H, d, *J* = 16.0 Hz, -CH = CHCO-), 5.29 (1H, br s, H-12), 4.63 (1H, t, *J* = 8.0 Hz, H-3α), 2.83 (1H, dd, *J* = 10.4, 3.2 Hz, H-18), 1.14 (3H, s, CH_3_), 0.97 (3H, s, CH_3_), 0.93 (6H, s, CH_3_), 0.91 (6H, s, CH_3_), 0.77 (3H, s, CH_3_). ^13^C-NMR (100 MHz, CDCl_3_): *δ* 183.78, 166.67, 143.59, 139.68, 136.75, 130.70, 128.23, 128.01, 122.57, 117.60, 80.98, 55.33, 47.55, 46.53, 45.83, 41.56, 40.92, 39.28, 38.09, 37.94, 37.00, 33.78, 33.05, 32.53, 32.43, 30.66, 28.09, 27.66, 25.91, 23.63, 23.57, 23.40, 22.88, 18.19, 17.17, 16.82, 15.40. ESI-MS: *m/z* [M + Cl]^−^ 591.4.

### 3.23. Acidic Cleavage at Site B of Resin ***14*** with TFE

Resin **14** (42.6 mg, 0.0138 mmol) was mixed with a solution of AcOH/TFE/CH_2_Cl_2_ (1.0 mL, v/v/v = 1:2:7). And the mixture was stirred at room temperature for 2 h before the resin was filtered and washed with MeOH and CH_2_Cl_2_. The filtrate was concentrated *in vacuo*, and the residue was purified by column chromatography on silica gel (CHCl_3_-MeOH = 10:1) to afford product **16** (7.7 mg, 89.1%) and unreacted substrate **11a** (0.8 mg). 

*[2-(2-Aminoethoxy)ethoxy]methyl 3β-[(E)-(2-thiophene)acryloxyl]-12-en-28-oate* (**16**): ^1^H-NMR (300 MHz, CDCl_3_): *δ* 7.75 (1H, d, *J* = 15.6Hz, -CH = CHCO-), 7.35 (1H, d, *J* = 4.8 Hz, H-3 of thiophene), 7.23 (1H, d, *J* = 2.7 Hz, H-5 of thiophene), 7.03 (1H, t, *J* = 3.9 Hz, H-4 of thiophene), 6.23 (1H, d, *J* = 15.9 Hz, -CH = CHCO-), 5.28 (3H, m, H-12, -COOCH_2_O-), 4.61 (1H, t, *J* = 7.8 Hz, H-3α), 4.32 (2H, br s, -NH_2_), 3.78 (2H, br s, -NCH_2_CH_2_O-), 3.63 (4H, br s, -OCH_2_CH_2_O-), 2.99 (2H, br s, -NCH_2_CH_2_O-), 2.86 (1H, d, *J* = 11.1 Hz, H-18), 1.14 (3H, s, CH_3_), 0.94 (3H, s, CH_3_), 0.92 (6H, s, CH_3_), 0.90 (6H, s, CH_3_), 0.74 (3H, s, CH_3_). ^13^C-NMR (75 MHz, CDCl_3_): *δ* 177.21, 166.63, 143.58, 139.64, 136.73, 130.68, 128.22, 128.00, 122.44, 117.57, 89.24, 80.91, 71.03, 69.95, 69.35, 55.30, 47.48, 46.89, 45.76, 41.70, 41.18, 39.33, 38.11, 37.90, 36.90, 33.79, 33.03, 32.68, 32.31, 30.65 (2C), 28.07, 27.58, 25.76, 23.58 (2C), 23.39, 22.91, 18.21, 17.03, 16.83, 15.38. ESI-MS: *m/z* [M + H]^+^ 710.4.

### 3.24. Cytotoxicity Assay

Cells grown in 96-well plates were treated with gradient concentrations of each compound for 48 h. Cells were fixed with 50% trichloroacetic acid and stained with 0.4% sulforhodamine B dissolved in 1% acetic acid. Cells were then washed with 1% acetic acid to remove unbound dye. The protein-bound dye was extracted with 10 mM Tris base to determine the optical density at 564 nm wavelength using a SPECTRAmax PLUS 384 microplate spectrophotometer (Molecular Devices, Sunnyvale, CA, USA). The percentage of cell survival as a function of drug concentration was plotted to determine the IC_50_ value, which stands for the drug concentration needed to kill cells by 50%.

### 3.25. Cell Migration Assay

Human umbilical vein endothelial cells grown in 24-well plates as confluent monolayers were mechanically scratched using a 20 µL pipette tip to create the wound. Cells were washed with phosphate-buffered saline to remove the debris, and complete culture media were then added to allow wound healing. Phase contrast images of the wound were taken at three random locations first immediately after wounding and then at the same location after 24 h to examine wound closure by migrating cells. Cells migrated into the wound area were then counted, and the extent of wound closure was determined.

## 4. Conclusions

In summary, a hydrophilic amino-terminated poly(ethylene glycol)-type dual linker for the solid phase synthesis of oleanolic acid derivatives using trityl chloride resin was designed and synthesized for the first time. Although the sensitivity of the linker to acids limits its applications, careful planning of the synthetic approach may overcome this problem. Following the successful solid-phase synthesis of model compounds **15** and **16**, the preparation of an OA analog library and the potential of this dual linker in reaction monitoring and target identification are under investigation in our lab.
